# Supplementary material for: Inhibited KdpFABC transitions into an E1 off-cycle state
Source: eLife. 2022 Oct 18;11:e80988. doi: 10.7554/eLife.80988 (PMC9651954; doi:10.7554/eLife.80988)
Supplement: Table 1—source data 1. [file elife-80988-table1-data1.docx]

**Table 1 – Source Data 1: Cryo-EM data collection, refinement, and validation statistics**

|  | **KdpFABC**  **E1·ATP_early_**  **EMD-14913**  **[7ZRG]** | **KdpFABC**  **E1P·ADP**  **EMD-14917**  **[7ZRK]** | **KdpFABC**  **E1P tight**  **EMD-14912**  **[7ZRE]** | **KdpFABC**  **E1P tight**  **(VO_4_^3-^)**  **EMD-14911**  **[7ZRD]** | **KdpFABC**  **E2P**  **(VO_4_^3-^)**  **EMD-14347** | **KdpFAB_S162A_C**  **E1P·ADP**  **EMD-14919**  **[7ZRM]** | **KdpFAB_S162A_C**  **E2P**  **EMD-14918**  **[7ZRL]** | **KdpFAB_D307N_C**  **E1 apo tight**  **EMD-14914**  **[7ZRH]** | **KdpFAB_D307N_C**  **E1 apo open 1**  **EMD-14915**  **[7ZRI]** | **KdpFAB_D307N_C**  **E1 apo open 2**  **EMD-14916**  **[7ZRJ]** |
| --- | --- | --- | --- | --- | --- | --- | --- | --- | --- | --- |
| **Data collection**  **and processing** |  |  |  |  |  |  |  |  |  |  |
| Magnification | 49,407 | 49,407 | 49,407 | 49,407 | 49,407 | 49,407 | 49,407 | 49,407 | 49,407 | 49,407 |
| Voltage (keV) | 200 | 200 | 200 | 200 | 200 | 200 | 200 | 200 | 200 | 200 |
| Electron exposure (e^-^/Å^2^) | 52 | 52 | 52 | 52 | 52 | 52 | 52 | 52 | 52 | 52 |
| Defocus range (µm) | -0.5 to -2.0 | -0.5 to -2.0 | -0.5 to -2.0 | -0.5 to -2.0 | -0.5 to -2.0 | -0.5 to -2.0 | -0.5 to -2.0 | -0.5 to -2.0 | -0.5 to -2.0 | -0.5 to -2.0 |
| Pixel size (Å) | 1.012 | 1.012 | 1.012 | 1.012 | 1.012 | 1.012 | 1.012 | 1.012 | 1.012 | 1.012 |
| Symmetry imposed | C1 | C1 | C1 | C1 | C1 | C1 | C1 | C1 | C1 | C1 |
| Initial particle images (no.) | 1,128,433 | 1,128,433 | 1,128,433 | 164,891 | 164,891 | 287,232 | 287,232 | 728,674 | 728,674 | 728,674 |
| Final particle images (no.) | 76,121 | 257,675 | 114488 | 74,927 | 13,508 | 58,243 | 46,904 | 88,852 | 75,711 | 47,981 |
| Map resolution (Å) | 3.5 | 3.1 | 3.4 | 3.3 | 7.4 | 3.7 | 4.0 | 3.4 | 3.5 | 3.7 |
| FSC threshold | 0.143 | 0.143 | 0.143 | 0.143 | 0.143 | 0.143 | 0.143 | 0.143 | 0.143 | 0.143 |
| Map resolution range (Å) | 3.3-6.5 | 3.0-5.5 | 3.1-5.0 | 3.1-5.0 | N/A | 3.5-6.5 | 3.8-7.0 | 3.3-5.5 | 3.4-6.5 | 3.6-6.5 |
|  |  |  |  |  |  |  |  |  |  |  |
| **Refinement** |  |  |  |  |  |  |  |  |  |  |
| Initial model used  (PDB code) | 7NNL | 7NNL | 6HRA | 6HRA | N/A | 7NNL | 6HRB | 6HRA | 6HRA | 6HRA |
| Model resolution (Å) | 3.7 | 3.6 | 3.6 | 3.5 | N/A | 4.0 | 4.1 | 3.5 | 3.6 | 3.7 |
| FSC threshold | 0.5 | 0.5 | 0.5 | 0.5 | N/A | 0.5 | 0.5 | 0.5 | 0.5 | 0.5 |
| Model resolution range (Å) | 80-3.5 | 80-3.1 | 80-3.4 | 80-3.3 | N/A | 80-3.7 | 80-4.0 | 80-3.4 | 80-3.5 | 80-3.7 |
| Sharpening B-factor (Å^2^) | -132 | -122 | -134 | -55 | -195 | -123 | -160 | -113 | -117 | -119 |
| Model composition |  |  |  |  |  |  |  |  |  |  |
| Non-hydrogen atoms | 11097 | 11103 | 11071 | 11072 | N/A | 10890 | 10782 | 11065 | 11065 | 11065 |
| Protein residues | 1456 | 1456 | 1456 | 1456 | N/A | 1456 | 1456 | 1456 | 1456 | 1456 |
| Ligands | K: 7 | K: 13 | K: 9 | K: 9 | N/A | K: 5 | K: 1 | K: 7 | K: 7 | K: 7 |
|  | CDL: 2 | CDL: 2 | CDL: 2 | CDL: 2 | N/A | ADP: 1 |  | CDL: 2 | CDL: 2 | CDL: 2 |
|  | ATP: 1 | ADP: 1 |  | VO4: 1 |  | MG: 1 |  |  |  |  |
|  |  | MG: 1 |  |  |  |  |  |  |  |  |
| B factors (Å^2^) |  |  |  |  |  |  |  |  |  |  |
| Protein | 55.0 | 54.3 | 50.2 | 23.8 | N/A | 54.8 | 69.9 | 33.0 | 52.5 | 47.5 |
| Ligand | 54.8 | 55.6 | 40.1 | 25.9 | N/A | 116.6 | 64.4 | 29.6 | 49.5 | 37.8 |
| R.m.s. deviations |  |  |  |  |  |  |  |  |  |  |
| Bond lengths (Å) | 0.006 | 0.006 | 0.007 | 0.006 | N/A | 0.004 | 0.005 | 0.004 | 0.004 | 0.005 |
| Bond angles (°) | 0.849 | 0.877 | 0.976 | 0.893 | N/A | 0.838 | 0.908 | 0.828 | 0.820 | 0.908 |
| Validation |  |  |  |  |  |  |  |  |  |  |
| MolProbity Score | 1.74 | 1.72 | 1.70 | 1.53 | N/A | 1.68 | 1.77 | 1.73 | 1.68 | 1.80 |
| Clash score | 7.24 | 8.04 | 7.79 | 5.88 | N/A | 6.82 | 8.17 | 7.43 | 6.90 | 9.34 |
| Poor rotamers, % | 0.00 | 0.00 | 0.00 | 0.00 | N/A | 0.00 | 0.00 | 0.00 | 0.00 | 0.00 |
| Ramachandran plot |  |  |  |  |  |  |  |  |  |  |
| Favored (%) | 95.02 | 95.91 | 95.98 | 96.68 | N/A | 95.64 | 95.26 | 94.43 | 95.71 | 95.64 |
| Allowed (%) | 4.98 | 4.09 | 4.02 | 3.32 | N/A | 4.36 | 4.74 | 4.57 | 4.29 | 4.36 |
| Outliers (%) | 0.00 | 0.00 | 0.00 | 0.00 | N/A | 0..00 | 0.00 | 0.00 | 0.00 | 0.00 |
